# Complete Genome Sequence of Four Strains of *Leptospira borgpetersenii* serovar Hardjo isolated from Cattle in the Central United States

**DOI:** 10.7150/jgen.69822

**Published:** 2022-02-14

**Authors:** Ellie J. Putz, Darrell O. Bayles, David P. Alt, Jarlath E. Nally

**Affiliations:** Infectious Bacterial Disease Research Unit, USDA Agriculture Research Service, National Animal Disease Center, Ames, IA, USA.

**Keywords:** *Leptospira borgpetersenii*, leptospirosis, bovine, PanOCT/PanACEA

## Abstract

Pathogenic species of *Leptospira* cause leptospirosis, a global zoonotic disease affecting humans and all major livestock species. Cattle act as a reservoir host for *L*. *borgpetersenii* serovar Hardjo which colonize the kidneys and reproductive tract from which they are excreted and transmitted to other cattle via urine, semen or uterine discharges. Bovine leptospirosis results in reproductive failure, abortion, stillbirth and loss of milk production, and is an occupational risk for those working with infected animals. A recent study determined that 7.2% of cattle from an abattoir in the central United States were actively shedding pathogenic *Leptospira*. Here, we report and compare the complete genome sequence of four recent isolates of *L*. *borgpetersenii* serovar Hardjo designated strain TC112, TC147, TC129, and TC273.

## Introduction

Leptospirosis is a global zoonotic disease caused by bacteria of the genus *Leptospira*. Over one million people suffer acute leptospirosis each year, and almost 60,000 people die 1. Leptospirosis is also a significant cause of morbidity and mortality in agricultural livestock animals since infected animals can present with acute disease as well as abortion, reproductive failure and weak offspring 2. Transmission of leptospires is maintained by reservoir hosts of infection, which shed leptospires exposing other hosts directly via urine, semen, and uterine discharges, or indirectly via contamination of moist environments 3. *Leptospira* are classified into 38 pathogenic species (clade P1 and P2) and comprise hundreds of serovars in multiple serogroups 4, 5. Multiple serovars of *L*. *interrogans* are the leading global cause of leptospirosis in humans. However, *L*. *borgpetersenii* serovar Hardjo is the leading global cause of bovine leptospirosis, with cattle serving as the primary reservoir host 2. The genome of *L*. *borgpetersenii* is ~700kb smaller than that of *L*. *interrogans* and is hypothesized to be undergoing genome reduction 6.

Bovine leptospirosis is endemic in the United States 7-9. During a recent survey in cattle from an abattoir in the Midwestern United States, it was found that 7.2% of animals were actively shedding *L. borgpetersenii* serovar Hardjo, by fluorescent antibody testing, PCR and culture of urine 8, 10. Four isolates (designated strains TC112, TC129, TC147, and TC273) cultured from bovine urine during that study have now been fully sequenced and are compared herein. All genome sequence information is available on GenBank (https://www.ncbi.nlm.nih.gov/genbank/), BioProject PRJNA759631. Accession numbers for chromosome 1 and chromosome 2 for each of the four strains, as well as genome annotation features, are provided in **Table 1**.

All strains were cultured at 29 °C until they reached the mid-late log growth phase in HAN media 11. Genome sequencing on the Illumina platform was performed as previously described 8. For genome sequencing on the Nanopore platform, ten ml of culture was pelleted by centrifugation at 7,000 × *g*, for 20 min at 4 °C. The supernatant was removed, and the pellet resuspended in 1 ml Phosphate-buffered saline (PBS), placed in a 1.5 ml Protein LoBind tube (Eppendorf, Hamburg, Germany) and pelleted by centrifugation at 12,000 × *g*, for 10 min at 4 °C. The supernatant was removed, and the pellet resuspended in 20 µl PBS. DNA was purified from each of the pellets with the Circulomics Nanobind CBB Big DNA Kit (Circulomics, Baltimore, MD, USA) using the Gram-Negative Bacteria High Molecular Weight DNA Extraction protocol. Purified DNA was quantified using the Thermo Fisher Qubit™ dsDNA BR Assay Kit (Thermo Fisher Scientific, Waltham, MA) and evaluated for quality and purity using a NanoPhotometer Pearl (Implen, Inc. Westlake Village, CA). Libraries for strains TC129 and TC273 were prepared using the ligation sequencing kit, (SQK-LSK-109), multiplexed using native barcoding expansion kit (EXP-NBD104) and sequenced using flow cell type R9.4.1 on a GridION 5X instrument (Oxford Nanopore, Oxford Science Park, UK). Libraries for strains TC147 and TC273 were prepared using the rapid barcoding kit (SQK-RBK004) and sequenced using the flongle flow cell type FLO-FLG001 on an MK1c instrument (Oxford Nanopore, Oxford Science Park, UK). Genome assembly and error correction was accomplished with Unicycler v. 0.4.4 (TC129 and TC273) and v. 0.4.7 (TC112 and TC147) 12. Oxford Nanopore and unpaired Illumina HiSeq reads were used with the Unicycler default settings except that a *Leptospira* ParA protein sequence was added to the start genes database to ensure that the small chromosome would be rotated to start at the *parA* gene. The genome annotation was completed by the NCBI Prokaryotic Genome Annotation Pipeline (PGAP) 13.

Comparative analysis of the complete genomes for each of the four recent *L*. *borgpetersenii* serovar Hardjo TC strains was performed against the complete genome of another bovine isolate of *L*. *borgpetersenii* serovar Hardjo; strain Hb15B203 (HB203) which was originally isolated from a 10-year old dairy cow from Kansas over 30 years ago 7. Since its isolation, strain HB203 has been utilized extensively as a challenge isolate in animal work for the Infectious Bacterial Disease Research unit (ARS, USDA) and its complete genome is also available on GenBank (BioProject: PRJNA384237) 14-18.

**Figure 1:**
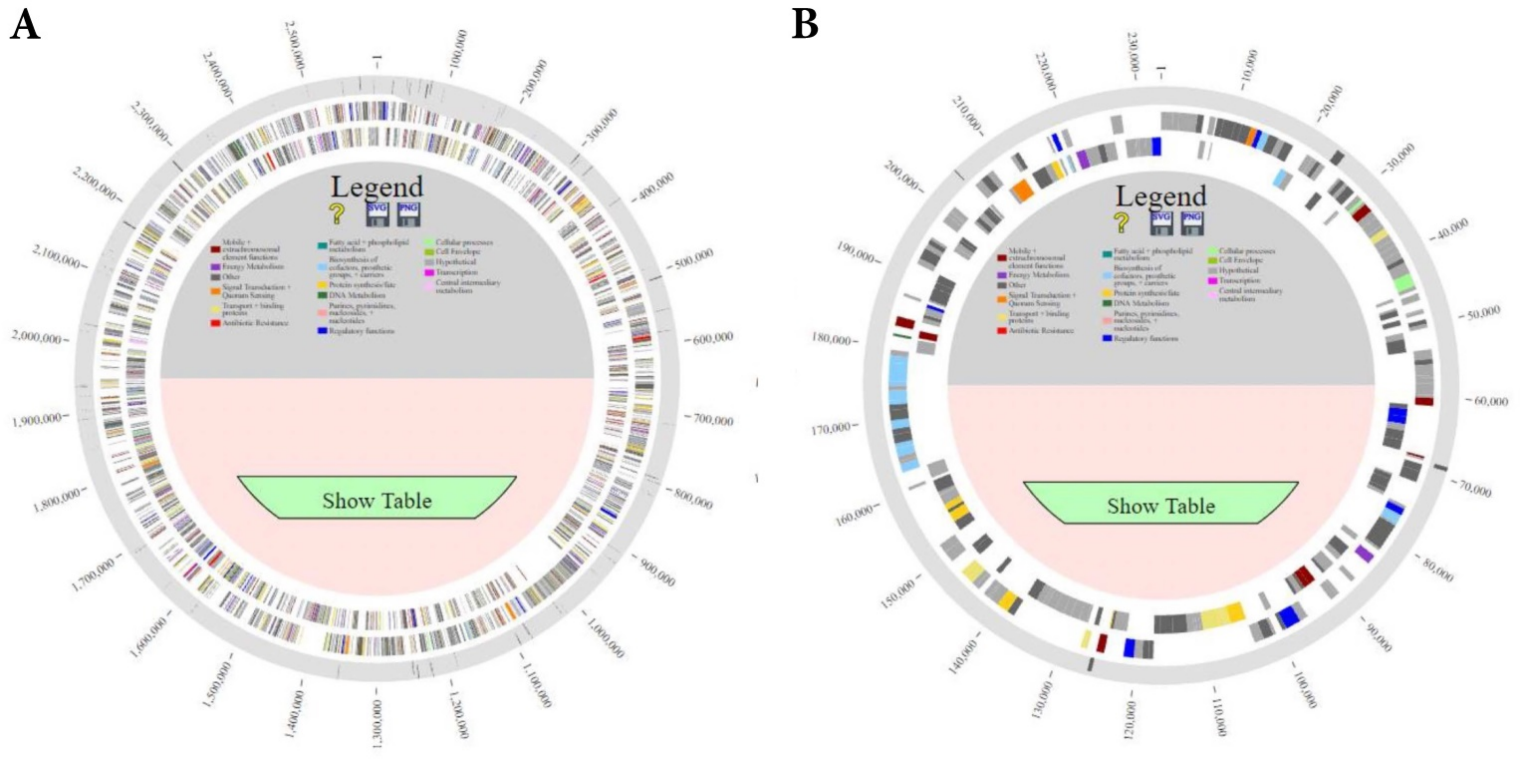
** Comparison of the genomes of *L. borgpetersenii* serovar Hardjo strains TC112, TC129, TC147, TC273 and HB203 with PanACEA.** Shown are **(A)** chromosome 1 and** (B)** chromosome 2. Using HB203 as the reference, the light gray color depicts core conserved regions and the dark gray lines within outermost circle depict variable regions between strains.

Comparison of all genomes using PanOCT (pan-genome ortholog clustering tool) and PanACEA (Pan-genome Atlas with Chromosome Explorer and Analyzer) identified differences in gene coding regions of the complete genome of strain HB203 and the more recently isolated strains TC112, TC129, TC147, and TC273 19, 20, **Figure 1**. In total, strain HB203 contained 206 genes not identified in any of the more recent TC strains. Conversely, 74 genes were identified in TC112, 90 genes in TC129, 88 genes in TC147 and 86 genes in TC273 that were not identified in strain HB203. A complete list of matching genes among all strains is provided in **Supplemental Table 1**. Of note, some variances in the PGAP pipeline's annotation of open reading frames (ORFs) for each of the TC strains were identified; For example, TC129 contained 14 small (averaging 57 amino acid) CDS which were not annotated in TC112 yet there are corresponding potential open reading frames of exactly the same size and with the same predicted amino acid for each one. After removal of these small *ab initio* gene calls from analysis, as listed **Supplementary Table 2**, and removal of some additional annotation calls for which homologous DNA was found in all TC strains (highlighted in yellow in supplementary Table 1), there were 71 genes identified in all four TC strains that had no homologous gene in HB203.

Globally, bovines are a reservoir host for *L*. *borgpetersenii* serovar Hardjo which are highly fastidious 2; a limited number of closed genomes have been sequenced but are poorly annotated. Of the 206 genes identified by PanOCT/PanACEA as unique to strain HB203, 134 (>65%) are annotated as encoding hypothetical proteins. Similarly, of the 71 genes unique to the TC strains, 32 (>45%) encode hypothetical proteins. As additional genomes are sequenced, comparative genome analyses with other strains obtained from different parts of the world, such as strain L550 in Australia 6, and separated by time, can provide insights into how this species and serovar continues to evolve and persistently infect cattle despite the availability of bacterin vaccines.

Genome comparisons using the single nucleotide polymorphisms (SNPs) calling capabilities of Mauve software 21 (Mauve development snapshot 2015-02-13; http://darlinglab.org/mauve/mauve.html) identified just 226 SNPs between the four novel TC strains and HB203. The SNP differences and their position in the respective genome are provided in **Supplemental Table 3**.

Collectively, these data demonstrate that the genomes of *L. borgpetersenii* serovar Hardjo strains TC112, TC129, TC147, and TC273 are highly similar to each other and to strain HB203 even though HB203 was isolated approximately 30 years earlier. Bovine leptospirosis due to *L*. *borgpetersenii* serovar Hardjo is a global disease. This work provides four additional complete genomes of recent bovine isolates of *L*. *borgpetersenii* serovar Hardjo obtained from U.S. cattle which will facilitate continued comparative genome analysis with other serovar Hardjo isolates, separated over time and geography, to identify conserved genome features, understand evolutionary traits that predispose serovar Hardjo to persistent carriage in cattle and zoonotic transmission, and gain additional insights into pathogenic mechanisms of infection.

## Supplementary Material

Supplementary tables.

## Figures and Tables

**Table 1:** Genome annotation of *L. borgpetersenii* serovar Hardjo strains

	TC112	TC129	TC147	TC273	HB203*
Accession Number (Chromosome 1 & 2)	CP084036 CP084037	CP084038 CP084039	CP084040 CP084041	CP084042 CP084043	CP021412 CP021413
Chromosome 1 (bp)	3,585,599	3,585,597	3,585,599	3,585,577	3,589,981
Chromosome 2 (bp)	317,347	317,349	317,349	317,347	317,347
G+C %	40	40	40	40	40
Genes (total)	3,389	3,403	3,403	3,403	3,539
CDSs (total)	3,345	3,359	3,359	3,359	3,495
Genes (coding)	2,945	2,962	2,962	2,958	3,079
CDSs (with protein)	2,945	2,962	2,962	2,958	3,079
Genes (RNA)	44	44	44	44	44
rRNAs (5S, 16S, 23S)	1, 2, 2	1, 2, 2	1, 2, 2	1, 2, 2	1, 2, 2
tRNAs	37	37	37	37	37
ncRNAs	2	2	2	2	2
Pseudo Genes (total)	400	397	397	401	416

*Strain of *L*. *borgpetersenii* serovar Hardjo originally isolated from bovine over 30 years ago 7.
